# Situation Analysis of a New Effort of Community-Based Health Planning and Services (CHPS) for Maternal Health in Upper West Region in Rural Ghana

**DOI:** 10.3390/ijerph20166595

**Published:** 2023-08-18

**Authors:** Chieko Matsubara, Maxwell Ayindenaba Dalaba, Laata Latif Danchaka, Paul Welaga

**Affiliations:** 1 Bureau of International Medical Cooperation, National Center for Global Health and Medicine, 1-21-1 Toyama, Shinjuku-ku, Tokyo 162-8655, Japan; 2 Centre for Non-Communicable Diseases Research, Institute of Health Research, University of Health and Allied Sciences, P.O. Box 31, Ho, Volta Region, Ghana; mdalaba@uhas.edu.gh; 3 Navrongo Health Research Centre, P.O. Box 114, Navrongo, Upper East Region, Ghana; 4 Wa Technical Institute, P.O. Box 238, Wa, Upper West Region, Ghana; 5 Department of Biostatistics and Epidemiology, School of Public Health, C.K. Tedam University of Technology and Applied Sciences, P.O. Box 24, Navrongo, Upper East Region, Ghana

**Keywords:** child birth, CHPS, midwife, cost of delivery, out-of-pocket payment, free maternal health policy, facility utilization, Upper West Region, Ghana

## Abstract

A free maternal health policy started in Ghana in 2008, however, health facility utilization is still low, and out-of-pocket payments (OOPPs) are putting households at risk of catastrophic expenditure. To improve this situation, some rural communities have assigned a midwife to a health post called community-based health planning and services (CHPS), where only assistant nurses are allocated. This study explored the effectiveness of the new approach in Upper West Region, Ghana. We conducted a cross-sectional study and interviewed women who gave birth in the last year. We systematically selected communities matched into four criteria: communities near CHPS (functional CHPS), communities near CHPS with a midwife (advanced CHPS), communities near a health centre, and communities without a health facility in their neighbourhood. In total, 534 women were interviewed: functional CHPS 104, advanced CHPS 131, near health centre 173, and no facility 126. About 78% of the women were 20 to 34 years old. About half of the women incurred OOPP, however, catastrophic payment (household spending > 5% of annual income) was significantly lower in advanced CHPS communities for normal delivery compared with the other three communities. The new local approach of assigning a midwife to CHPS functioned well, improving access to healthcare facilities for childbirth.

## 1. Introduction

An improvement in maternal mortality has been a priority agenda for sub-Saharan African countries, and many global, regional, and national initiatives have been established, and there has been significant progress since 2000. The maternal mortality worldwide declined by 34% from 2000 to 2020, from 342 deaths to 223 deaths per 100,000 live births; however, there were 545 maternal deaths per 100,000 live births in sub-Saharan Africa in 2020 [1]. This remains unacceptably high, and most of the maternal deaths could be prevented [2].

Access to affordable and quality healthcare services remains a challenge in many low-income countries, particularly in sub-Saharan Africa. Many preventable maternal deaths still happen across many low- and middle-income countries due to the lack of access and financial hardship women face in seeking healthcare [3]. The cost of healthcare often poses a significant burden on households in low- and middle-income countries. Out-of-pocket payments (OOPPs) are reported to be a major barrier to seeking maternal healthcare, especially for the poor, and can expose households to a risk of catastrophic expenditure and impoverishment [4,5,6].

Maternal mortality in Ghana, one of the sub-Saharan countries, has gradually declined from 484 deaths per 100,000 live births in 2000 to 308 deaths per 100,000 live births in 2017 [7]: coastal zone 336, middle zone 296, and northern zone 276, respectively, in 2017 [8]. Therefore, further improvements are necessary as they are in other sub-Saharan countries.

To address the challenges in seeking accessible, affordable, and quality healthcare services, community-based health planning and services (CHPS) was developed, and has been implemented since 1999. CHPS is a national strategy to deliver essential health services to rural communities, involving health planning and service delivery with community members [9,10,11]. One or two community nurse(s), called community health officers (CHOs), is/are assigned to CHPS. CHOs are government employees, trained for two years at community nurse school, and they live and work in the community [12]. They provide services to about 5000 people within a district’s border areas, called CHPS zones, supervised by public health nurses, physician assistants, and sub-district CHPS coordinators [12]. They regularly hold discussions with community members (community durbar) to set up community health planning and to implement the CHPS health service package based on the community’s health needs. Community members are required to support CHPS operations through volunteerism such as fetching water and mowing. CHPS is the frontline unit of healthcare delivery in rural areas and it provides mainly preventive care with some curative care services only for minor illness. CHOs provide antenatal, maternity, and postnatal care to ensure safe pregnancy and delivery. On the other hand, health centres provide basic curative care, disease prevention, maternal, and child health services [13]. Therefore, pregnant women receive antenatal and postnatal care at CHPS, but these women need to go to a health centre or hospital for their deliveries.

To further expand maternal healthcare services, the government has implemented the free maternal healthcare policy through the National Health Insurance Scheme (NHIS) since 2008 [14]. This policy accelerated pregnant women going to health facilities, however, the utilization rate of health facilities is lower in rural areas (68%) than in urban areas (90%) [8]. Furthermore, OOPPs are still reported under the free maternal healthcare policy [6,15], so the risk of catastrophic payments and impoverishment frightens poor households [6].

To break through this situation, some rural communities began a new maternal health system in collaboration with the local government in around 2014. This system, called advanced CHPS, involved the assignment of a midwife to a CHPS to support delivery at the CHPS compounds.

The aim of this study was to explore the effects of advanced CHPS on access to childbirth and the costs of childbirth.

## 2. Materials and Methods

### 2.1. Study Area

The study was conducted in the Upper West Region (UWR) in Ghana. The UWR is bordered by Burkina Faso to its north and west. The northern portion of UWR has a dry climate with yearly evaporation exceeding annual precipitation, whereas the southern portion has a tropical rainy climate with a definite dry season [16]. Its population is 901,502 (2021), and the population density is low [16]. In this region, subsistence agriculture is the mainstay of the population and the region is one of the poorest in Ghana. The major ethnic groups in the region are the Dagaaba, Sissala, and Wala [17]. The UWR is home to 242 health facilities that offer a range of services. Three district government hospitals, one regional hospital, two hospitals run by the Christian Health Association of Ghana (CHAG), and three private hospitals are among them. The remaining facilities consist of 4 maternity homes, 147 CHPS compounds, 66 health centres, and 10 clinics [17].

### 2.2. Study Design

We conducted a cross-sectional study between January and April 2016 with a quantitative approach used in data collection. A structured questionnaire was used to obtain data from women of reproductive age who gave birth between January and December 2015. If a woman gave birth to many children within this period, the cost of childbirth was collected on the most recent childbirth.

The structured questionnaire provided sections for socio-demographic characteristics, place of childbirth, cost of childbirth, and household wealth [17,18].

### 2.3. Sample Size

The sample size for this study was calculated by assuming that 50% of the women in the study districts would have given birth in 2015 [17,19,20] and with a 95% confidence level, and a design effect of 1.3 [21] to account for the clustering effect in the districts would require a sample size of 500. Adjusting for a non-response rate of 15% yielded a sample size of 575.

### 2.4. Sampling and Data Collection

We systematically selected communities matched to the criteria in three linguistic areas in Upper West: communities near CHPS (functional CHPS), communities near CHPS with a midwife (advanced CHPS), communities near a health centre, communities without a health facility.

**Figure 1:**
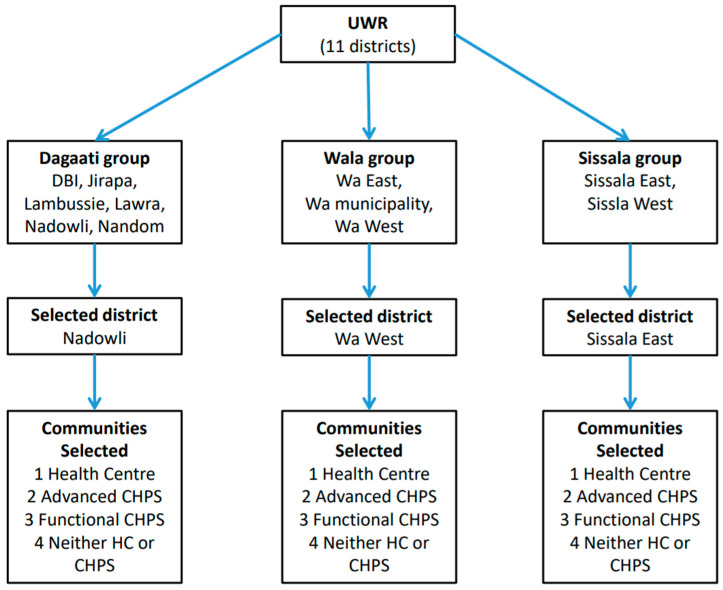
Sampling strategy.

We systematically selected the respondents using multistage sampling as shown in Figure 1. First, we divided the UWR’s 11 districts into three local linguistic groups as follows: (i) Dagaati group (Daffiama Bussie Issa (DBI) district, Jirapa district, Lambussie district, Lawra district, Nadowli district, and Nandom district); (ii) Wala group (Wa East district, Wa Municipality, and Wa West district); and (iii) Sissala group (Sissala East district and Sissala West district).

Next, we chose one district at random from each local linguistic group: Sissala East district to represent the Sissala ethnic group, Wa West district to represent the Wala group, and Nadowli district to represent the Dagaati group. Through this plan, the region was represented geographically and racially.

Thirdly, we chose four sub-districts within each district based on the following four criteria: a sub-district close to a CHPS (functional CHPS), a sub-district close to a CHPS with a midwife (advanced CHPS), a sub-district close to a health centre, and a sub-district with no nearby health facility. If more than one sub-district met a given requirement, we chose one at random. Then, we chose five communities from each sub-district at random.

Women who gave birth between January and December 2015 were the respondents in this study. By moving from one family to the next until they met their sample size, interviewers were given the assignment to the communities and instructed to find 39 respondents in each neighborhood. The interviewer spun a pen in the middle of the community to choose households for the interview. The first household was selected by going in the direction that the pen was pointing. Only one of the women who lived in the household who gave birth during the last year was randomly chosen for the interview. The interviewer then moved on to the following household. The interviewer repeated this process until they acquired the required number of respondents from the community.

Graduate-level interviewers conducted the interviews. The interviewees were chosen based on their familiarity with the chosen local area, their proficiency in the local language(s), and their prior data collection experience. The interviewers received training on the research protocol, the questionnaire, and policies, including those pertaining to obtaining informed consent for conducting interviews. Pre-testing was performed during the training to make sure the questionnaire’s questions were appropriate. All issues were fixed before the data collection.

### 2.5. Data Processing and Analysis

The data were gathered using a paper-based questionnaire. Then, using EPI Data 6.1 (The EpiData Association, Odense, Denmark), two data entry clerks double-entered the data. After entry, the data were cleaned and verified. In case there were any discrepancies between the records made by the two clerks, we fixed the data by comparing the source questionnaire.

Then, the data were imported to STATA version 16.0 (StataCorp LLC, Texas, USA) and SPSS version 27 (IBM Corp, New York, USA) for analysis. We cleaned up the data by identifying outliers and missing values in frequencies and cross tabulations; we confirmed that the variables were consistent. Tables were made to show means, proportions, and frequencies. We estimated the cost of childbirth from the viewpoint of the households/mothers.

We calculated the total childbirth cost by summing up direct medical and non-medical costs [17]. The direct medical costs included OOPPs for registration cards, consultations, diagnoses (scanning and laboratory tests), medication, and medical equipment. The direct non-medical costs included OOPPs for travel to and from health facilities, informal payments, and other non-medical expenses including disinfection, a rubber bed spread, cotton, gauze, a delivery pad, spirit, and so on. Informal payments are sums of money, either in cash or in kind, given to healthcare providers by mothers or their family members in addition to the required contribution [22].

The anticipated cost of agricultural product yields, such as crops and poultry, as well as business salaries and wages, investment profits, and remittance money were used to compute the annual income of households.

Catastrophic payments happen when the cumulative OOPPs for healthcare surpass a certain percentage of a household’s resources (income or expenditures) [14,23,24]. Generally, this percentage is between 5 and 40%. For the purposes of this study, we selected a 5% threshold for catastrophic payment. Accordingly, catastrophic payment was supposed to happen when the cost of childbirth was less than 5% of the household’s annual income. Since monthly income would cause a significant disparity between months when key crops were harvested and months when they were not, we used annual income instead. According to the average currency rate used in 2018 (GHC 1 = USD 0.2 as of 5 October 2018 [25]), all study expenditures were recorded in Ghana Cedis (GHC), and the results are given in USD.

### 2.6. Ethical Consideration

The Navrongo Health Research Centre, Upper East, Ghana (Approval ID: NHRCIRB232), and the National Centre for Global Health and Medicine (NCGM), Japan, (Approval ID: NCGM-G-0020510-00) Research Ethics Committees evaluated and approved the study protocol, respectively. All respondents received an explanation of the study’s methodology in either the Dagaati, Sissala, or Wala language, and written informed consent was collected in either of their preferred languages. Illiterate respondents signed the consent form with their thumbs printed on the informed consent form written in their preferred local language.

## 3. Results

### 3.1. Background Characteristics of Respondents

The background characteristics of respondents are shown in Table 1. The total number of respondents included in the analysis was 534. Approximately 45% of the respondents identified as Dagaati, 32% as Sissala, and 18% as Wala. The respondents, or 78% of them, ranged in age from 20 to 34. A total of 60.8% of them had never attended school, 19.5% had finished elementary school, and 0.6% had completed college or university. The majority of responders (80.0%) worked as farmers. At the time of the interview, almost 85% of the respondents had signed up for the NHIS. The estimated median household monthly income was GHS 1176 (IQR: 350 to 3168).

### 3.2. Choice of Place of Childbirth

Table 2 presents the choice of place of normal childbirth by four criteria of health facility areas. The appropriateness of the place of childbirth is shown by the coloured highlights: a green highlight shows that the mother gave birth at a proper (expected) place, a yellow highlight shows that the mother gave birth at a proper place but outside (crossing) the residential area, a red highlight shows that the mother gave birth at an unproper place. The utilization of a CHPS compound for delivery was highest in advanced CHPS communities (49%) and also functional CHPS communities (42%), even though women living in functional CHPS communities needed to go to a health centre or a district hospital where qualified medical staff would support their deliveries.

The percentage of childbirths in the proper place, shown by summing up the percentages labelled green, was highest in health centre communities (72.6%), followed by advanced CHPS communities (71.0%), neither health centre nor CHPS communities (66.1%), and functional CHPS (43.3%).

On the other hand, there was no statistically significant difference in home delivery (unproper place) among the four types of communities (14.4 to 19.9%). A uniqueness was found that about 10% of pregnant women in communities near health centres gave birth at CHPS, which is a lower level of health facility than a health centre.

### 3.3. Cost of Childbirth

Table 3 shows the childbirth cost for normal birth by the four criteria of health facility areas. About half of pregnant women were compelled to pay some amount to buy medicines, disinfectants, sanitary pads, and so on. It is not shown in the table, but most mothers who gave birth at home did not pay any costs to birth attendants, and some of the mothers answered that they gave crops, ground nuts, and so on, to birth attendants as gifts. Most incurred more costs at the advanced CHPS (GHS 32.3) compared to the other places of childbirth, while the least cost was incurred at the health centre (GHS 25.4). We do not have information on birth attendants for each respondent.

### 3.4. Catastrophic Payment

Table 4 reveals catastrophic payments for normal childbirth by residential area. Catastrophic payment (household spent more than 5% of annual income) was significantly lower in advanced CHPS communities compared to the other three areas (*p* = 0.006).

## 4. Discussion

The aim of this study was to examine the effects of advanced CHPS on access to childbirth and the costs of childbirth. This study found that a new local health system to assign a midwife on CHPS (advanced CHPS) functioned well in reducing the rate of catastrophic payments for normal childbirth compared to other residential criteria (near health centre, near functional CHPS, and neither health centre nor CHPS); the burden of OOPP and the catastrophic payment due to childbirth expenditure were reported as an emerging issue under the free maternal healthcare policy in previous studies [6,15].

However, this study shows that the OOPP for normal childbirth was still common in Upper West Region, even under the free maternal care policy and with a high coverage of health insurance (85.2%). A previous study also revealed that 21% of households spent more than 10% of their monthly income on child birth in Upper West Region, and the burden of OOPP on child birth was more serious among poor households [6]. This previous study also showed that the cost of home delivery was lower than that of CHPS, health centres, and hospitals. Therefore, if catastrophic payment due to childbirth costs cannot be stopped, poor households might deliver their baby at home, which is the least expensive option even though it is not a safe place for childbirth. This might be one of the reasons why about 15% of mothers still gave birth to their last baby at home regardless of residential zone. The capacity of health facilities (health centre and CHPS) needs to be strengthened with required medicines and equipment to eliminate payments in order to promote the free maternal healthcare agenda.

Regarding health facility utilization, this study showed that women delivered their babies at a health facility within their CHPS zones or health policy zones; 62% of women delivered at a health centre in communities near health centres, 49% and 42% of women delivered at a CHPS in advanced CHPS communities and functional CHPS communities, respectively. A previous study showed that CHPS service utilization is higher where satisfaction is higher [26], on the other hand, a negative attitude, inadequacy, and unavailability of CHPS staff are reported as challenges by another study conducted in the Volta region [11], which is one of the regions of the coastal area, where health facility utilization is lowest in Ghana [8]. The result of this study may latently indicate satisfaction of women living in a CHPS zone.

During the data collection, although we did not provide a space for comments in the questionnaire, we often heard comments from respondents in the advanced CHPS communities that they respect midwives at advanced CHPS as medical persons who were qualified to support women’s deliveries and their kind and empathetic attitudes towards women.

Midwives and CHOs can focus on the CHPS area and make CHPS health service plans based on the health needs of the community, including pregnant women. On the other hand, medical doctors and registered nurses may be fully respected as medical experts by the community, however, they may have to cover and supervise not only communities near the health centre but also communities under CHPS and communities with neither a health centre nor CHPS. For this reason, mothers living in advanced CHPS areas might be more properly recognised and want to be cared for by CHPS midwives. If more midwives are assigned to functional CHPS, more women could be cared for properly.

### Limitations of the Study

Recall bias is a possible limitation given that respondents were asked to recall expenditure over a one year period. Because respondents merely verbally reported their expenses rather than providing receipts, the reported spending may have been overstated or understated. However, we employed a probing technique that may reduce this bias, and we do not anticipate that it will have had a negative impact on the study’s findings.

In addition, this study did not assess the place of delivery and the cost of delivery disaggregated by the three language groups in the study area. Further studies would be desirable in the future to show local characteristics in more detail.

Another limitation is that this study did not include a qualitative approach. A mixed method would provide richer data for this study. However, the quantitative questionnaire was well developed and the results of this study captured components that are relevant to this study.

## 5. Conclusions

A new local health system, advanced CHPS areas, where a midwife(s) is assigned, was successful in showing statistically lower catastrophic household payments (*p* < 0.05) for normal childbirth compared with other health facility settings (health centre areas, functional CHPS areas, and neither health centre nor CHPS areas) in rural Ghana. About half of women delivered their babies at advanced CHPS in advanced CHPS communities. However, mothers still paid OOPP under the free maternal health service policy in every category of communities. In order to prevent catastrophic payment, particularly in poor households’ due to the OOPP for child birth, and to raise health facility delivery, it would be effective to expand the advanced CHPS approach as well as strengthening the capacity of health facilities. If the OOPP and the catastrophic payment were reduced through the advanced CHPS approach, access to healthcare facilities for child birth would be improved and the home delivery rate would be reduced, providing better maternal healthcare.

## Figures and Tables

**Table 1:** Background of respondents interviewed (*n* = 534).

Variable	Number	%
Age Group		
15–19	18	3.4
20–34	416	77.9
35+	100	18.7
Ethnicity		
Wala	96	18.0
Sissala	170	31.8
Dagaati	242	45.3
Other	26	4.9
Education		
Never been to school	324	60.8
Primary school	104	19.5
Junior high	74	13.9
Senior high	28	5.3
University/College	3	0.6
Occupation		
Farmer	427	80.0
Industrial worker	2	0.4
Home industry	52	9.7
Market vender	25	4.7
NGO/Private employee	2	0.4
Shop/Restaurant	4	0.8
Civil servant	2	0.4
No income	16	3.0
Student	4	0.8
NHIS status of respondents		
Yes	449	85.2
No	78	14.8
Household annual income		
Median (IQR)	GHS 1176	(350–3168)

GHS 1 = USD 0.20 as of 5 October 2018.

**Table 2:** Place of delivery for normal birth.

		Frequency	%
Near Health Centre (*n* = 160)	Home (No visit)	28	17.5
	CHPS	16	10.0
	Health Centre	99	61.9
	District Hospital	7	4.4
	Others	10	6.3
Near Advanced CHPS (*n* = 131)	Home (No visit)	26	19.9
	CHPS	64	48.9
	Health Centre	12	9.2
	District Hospital	19	14.5
	Others	10	7.6
Near Functional CHPS (*n* = 104)	Home (No visit)	15	14.4
	CHPS	44	42.3
	Health Centre	30	28.9
	District Hospital	10	9.6
	Others	5	4.8
Neither Health Center Nor CHPS (*n* = 139)	Home (No visit)	21	15.1
	CHPS	16	11.5
	Health Centre	52	37.4
	District Hospital	38	27.3
	Others	2	1.4
CHPS: Community-based Health Planning and Services			
	Proper place		
	Crossing		
	Unproper place		

**Table 3:** Delivery care cost for normal birth (GHS). GHS 1 = USD 0.20 as of 5 October 2018.

		Number That Paid (%)	Average Cost (SD)	Minimum Cost (GHS)	Maximum Cost (GHS)
Near Health Centre (*n* = 160)	Medical cost	68 (42.5)	15.3 (17.8)	1	85
	Non-medical cost	89 (55.6)	20.8 (53.3)	1	500
	Total	114 (71.3)	25.4 (48.6)	1	502
Near Advanced CHPS (*n* = 131)	Medical cost	39 (29.8)	28.8 (38.8)	2	220
	Non-medical cost	63 (48.1)	21.2 (27.9)	2	150
	Total	76 (58.0)	32.3 (39.9)	2	230
Near Functional CHPS (*n* = 104)	Medical cost	28 (26.9)	25.2 (25.5)	1	100
	Non-medical cost	68 (48.1)	22.2 (18.2)	2	73
	Total	72 (69.2)	30.8 (18.2)	1	140
Neither Health Center Nor CHPS (*n* = 139)	Medical cost	51 (36.7)	20.9 (27.9)	1	120
	Non-medical cost	100 (71.9)	21.2 (33.7)	2	224
	Total	105 (75.5)	30.3 (41.7)	3	254

CHPS: community-based health planning and services.

**Table 4:** Catastrophic payment ^¶^ for normal birth delivery care by residential area.

		Residential Area							
		Health Centre		Advanced CHPS		Functional CHPS		Neither Health Centre or CHPS	
		Freq.	%	Freq.	%	Freq.	%	Freq.	%
Catastrophic payment	Yes	20	14.0	6	5.0	16	18.8	22	19.1
	No	123	6.0	115	95.0	69	81.2	93	80.9

*p* = 0.006. ^¶^ Households spent more than 5% of their annual income to pay for normal birth delivery care.

## Data Availability

Parties who wish to access the data set should contact the corresponding author, Dr. Chieko Matsubara (c-matsubara@it.ncgm.go.jp), Bureau of International Medical Cooperation, National Center for Global Health and Medicine. She will respond individually to the requests for the dataset.
